# Right ventricular function and structure: results from the Multi-Ethnic Study of Atherosclerosis

**DOI:** 10.1186/1532-429X-11-S1-P110

**Published:** 2009-01-28

**Authors:** Harjit Chahal, Craig Johnson, Harikrishna Tandri, Aditya Jain, Gregory Hundley, Graham Barr, Steven Kawut, João Lima, David Bluemke

**Affiliations:** 1 Johns Hopkins University, Baltimore, MD USA; 2 University of Washington, Seattle, WA USA; 3 Wake Forest University Health Sciences, Winston-Salem, NC USA; 4 Columbia University, New York, NY USA

**Keywords:** Right Ventricle, Pearson Coefficient, Systolic Volume, Traditional Cardiovascular Risk Factor, Left Ventricle Mass

## Introduction

Recent studies of right ventricle (RV) structure and function have recognized the critical role of the RV in maintaining cardiac function. Previous studies on the RV have been limited by sample size and inadequate inclusion of participants from different races. The Multi-Ethnic Study of Atherosclerosis (MESA) is a large ongoing multi-center study that aims to investigate the subclinical progression of cardiovascular disease in an ethnically diverse clinically asymptomatic population.

## Purpose

Traditional cardiovascular risk factors have been extensively studied in relationship to the left ventricle (LV). MRI has not been previously used to determine if the RV is independent of these traditional risk factors. The purpose of this study was to assess the relationships of traditional cardiovascular risk factors to RV morphology and function and their relationship to the LV.

## Methods

Cardiac MRIs were performed on 5,004 participants without clinical cardiovascular disease at six field centers. 1572 randomly selected participants had complete interpretation of RV measures (46% men, mean age 61 ± 10 years). Endocardial margins of the RV were manually contoured on diastolic and systolic images. End-diastolic volume (EDV) and end-systolic volume (ESV) were calculated by using a summation of disks method ("Simpson's Rule"). Baseline variables included demographics and cardiac risk factors. Multivariate linear regression was performed for RV parameters versus traditional cardiovascular risk factors after adjusting for socio-demographic parameters. To evaluate intra-reader and inter-reader variability, 10% of the scans were selected randomly as re-reads (5% intra-reader and 5% inter-reader). Intra-class correlation coefficients (ICC) and Pearson coefficients were used to describe the reproducibility in RV measures.

## Results

**Figure 1:**
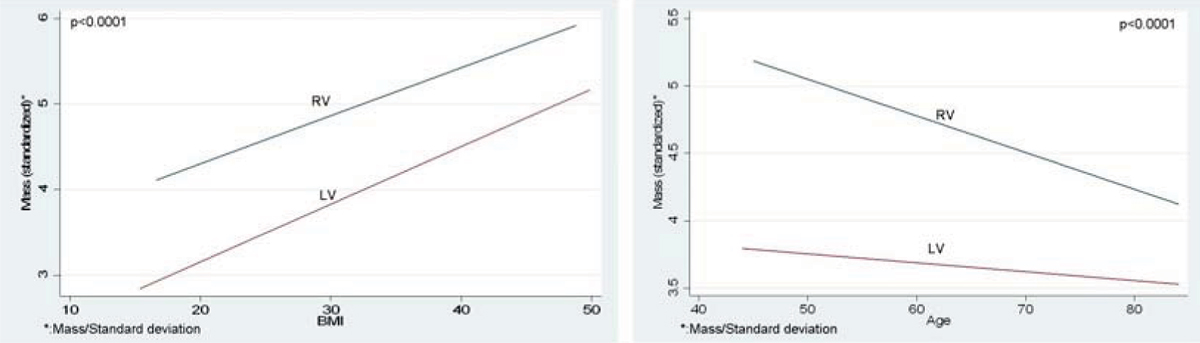
Figure 1

All RV parameters (mass, diastolic and systolic volumes) showed a negative association with age (p < 0.0001). After adjusting for body size, men had significantly higher RV mass and volumes than women. LV mass and volumes were positively associated with systolic blood pressure, BMI, smoking and diabetes and inversely to LDL cholesterol. Of these, BMI, age and gender were associated with RV mass (1.5 g per 5 kg/m^2^ p < 0.0001; -1.0 g/10 yrs p < 0.0001 and 2.3 g, p < 0.0001, respectively). Systolic blood pressure was positively associated with RV mass (+0.3 g per 21 mm Hg, p < 0.05) and stroke volume (+2.0 ml per 21 Hg, p < 0.01). The ICC and Pearson coefficients for end diastolic and end systolic volume ranged from 0.93–0.96 and 0.87–0.98, respectively, and for RV mass 0.87–0.93 and 0.78–0.87, respectively. See Figure 1.

## Conclusion

Age, gender and BMI were related to RV mass and volumes. Other than blood pressure, traditional cardiovascular risk factors (diabetes mellitus, smoking, alcohol consumption, LDL cholesterol) had little relationship to RV mass and volume in the MESA cohort.

